# Engineering Enhanced Immunogenicity of Surface-Displayed Immunogens in a Killed Whole-Cell Genome-Reduced Bacterial Vaccine Platform Using Class I Viral Fusion Peptides

**DOI:** 10.3390/vaccines14010014

**Published:** 2025-12-22

**Authors:** Juan Sebastian Quintero-Barbosa, Yufeng Song, Frances Mehl, Shubham Mathur, Lauren Livingston, Xiaoying Shen, David C. Montefiori, Joshua Tan, Steven L. Zeichner

**Affiliations:** 1 Department of Pediatrics, University of Virginia, Charlottesville, VA 22908, USA; pye9my@virginia.edu (J.S.Q.-B.); bxw6yz@virginia.edu (Y.S.); mathur.shubham1990@gmail.com (S.M.); fja9zg@virginia.edu (L.L.); 2 Department of Surgery, Duke University, Durham, NC 27710, USA; sxshen@duke.edu (X.S.); monte@duke.edu (D.C.M.); 3 Duke Human Vaccine Institute, Duke University, Durham, NC 27710, USA; 4 Antibody Biology Unit, Laboratory of Immunogenetics, National Institute of Allergy and Infectious Diseases, National Institutes of Health, Rockville, MD 20852, USA; joshuahoongyu.tan@nih.gov; 5 Department of Microbiology, Immunology, and Cancer Biology, University of Virginia, Charlottesville, VA 22908, USA

**Keywords:** vaccine platform, killed whole-cell, genome-reduced bacteria, autotransporter, bacterin

## Abstract

**Background/Objectives**: New vaccine platforms that rapidly yield low-cost, easily manufactured vaccines are highly desired, yet current approaches lack key features. We developed the Killed Whole-Cell/Genome-Reduced Bacteria (KWC/GRB) platform, which uses a genome-reduced Gram-negative chassis to enhance antigen exposure and modularity via an autotransporter (AT) system. Integrated within a Design–Build–Test–Learn (DBTL) framework, KWC/GRB enables rapid iteration of engineered antigens and immunomodulatory elements. Here, we applied this platform to the HIV-1 fusion peptide (FP) and tested multiple antigen engineering strategies to enhance its immunogenicity. **Methods**: For a new vaccine, we synthesized DNA encoding the antigen together with selected immunomodulators and cloned the constructs into a plasmid. The plasmids were transformed into genome-reduced bacteria (GRB), which were grown, induced for antigen expression, and then inactivated to produce the vaccines. We tested multiple strategies to enhance antigen immunogenicity, including multimeric HIV-1 fusion peptide (FP) designs separated by different linkers and constructs incorporating immunomodulators such as TLR agonists, mucosal-immunity-promoting peptides, and a non-cognate T-cell agonist. Vaccines were selected based on structure prediction and confirmed surface expression by flow cytometry. Mice were vaccinated, and anti-FP antibody responses were measured by ELISA. **Results**: ELISA responses increased nearly one order of magnitude across design rounds, with the top-performing construct showing an ~8-fold improvement over the initial 1mer vaccine. Multimeric antigens separated by an α-helical linker were the most immunogenic. The non-cognate T-cell agonist increased responses context-dependently. Flow cytometry showed that increased anti-FP-mAb binding to GRB was associated with greater induction of antibody responses. Although anti-FP immune responses were greatly increased, the sera did not neutralize HIV. **Conclusions**: Although none of the constructs elicited detectable neutralizing activity, the combination of uniformly low AlphaFold pLDDT scores and the functional data suggests that the FP region may not adopt a stable native-like structure in this display context. Importantly, the results demonstrate that the KWC/GRB platform can generate highly immunogenic vaccines, and when applied to antigens with well-defined native tertiary structures, the approach should enable rapidly produced, high-response, very low-cost vaccines.

## 1. Introduction

The development of effective vaccines has transformed global health, drastically reducing the burden of infectious diseases [1,2,3]. While individual vaccines specifically designed to induce protective immune responses against particular pathogens have played critical roles in protecting humanity against deadly diseases [1,4], many contemporary vaccine research efforts have been directed at developing ‘platform’ vaccine technologies that can be used repeatedly to make different vaccines that protect against many different pathogens [5,6,7]. An ideal vaccine platform should meet several key criteria. It must be extremely low cost, making it suitable for use in high-income countries, in low- and middle-income nations (LMICs), and in agriculture/One Health settings to prevent animal diseases and limit interspecies transmission. It should enable rapid production, allowing the development of a testable vaccine soon after antigen target identification, which is essential for pandemic and biothreat response and potentially for personalized cancer immunotherapy. The platform should also offer ease of manufacture, relying on inexpensive, widely available feedstocks and on existing manufacturing infrastructure that can be used with minimal modification. It must be distribution-friendly, providing strong thermal stability and a long shelf life, and ideally support non-parenteral administration. Finally, it should be reusable for multiple vaccines, avoiding issues such as anti-vector immunity that can arise with viral-vectored platforms. Despite enormous progress, no existing vaccine platforms adequately meet all of these ideals [4,7,8,9,10,11,12].

Live-attenuated recombinant viral vaccines, like recombinant vaccinia and adenoviruses, and viral-vectored vaccines, like adenovirus vector vaccines, can pose biosafety concerns, and can fail to induce broad protective responses. They also suffer from the potential that they may not function for more than a single antigen, due to anti-vector immunity [13,14,15,16]. Protein subunit vaccines can require difficult and expensive manufacturing processes, particularly for challenging-to-express proteins, such as those that tend to form aggregates. They often require powerful adjuvants and multiple doses to induce robust immunity [17,18,19]. Nanoparticle vaccines are another platform technology that can be difficult, complicated, and expensive to manufacture [20,21]. A more recent vaccine platform, mRNA vaccines, has produced important, safe, and effective vaccines that have significantly improved human health, with the added advantages of adaptability to multiple pathogens and exceptional versatility and potency. However, mRNA vaccines require complex manufacturing pipelines, costly feedstocks, a production step involving encapsulation into lipid nanoparticles, and strict cold-chain conditions. These requirements make it challenging to reduce costs and infrastructure needs to levels that would allow widespread use in low- and middle-income countries (LMICs) or in animal health applications [6,22,23,24,25]. Even with substantial improvements in costs and stability, it is unlikely that mRNA vaccines would find widespread use in One Health applications, where acceptable costs are substantially less than $1 per dose [26]. Many governments and non-governmental organizations have articulated a “100-day” goal for a platform that could yield a new vaccine for biothreat response, which is challenging for mRNA vaccines [27,28].

**Figure 1:**
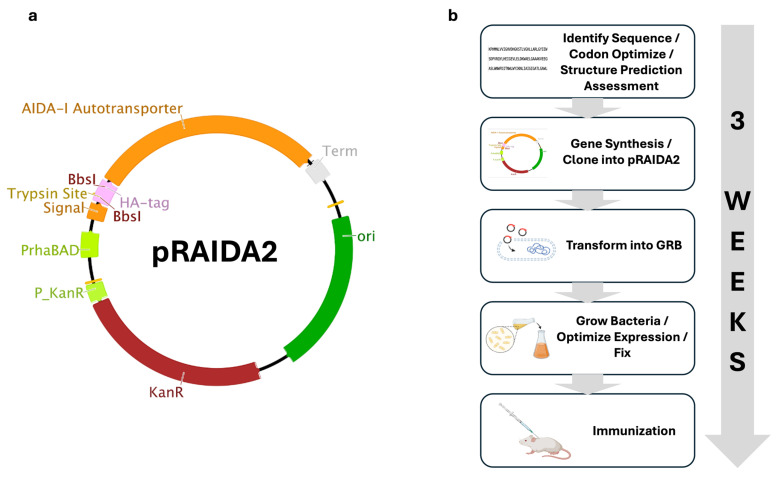
Vaccine platform design and implementation. (**a**) Schematic map of the synthetic plasmid pRAIDA2. Key features include a high-copy origin of replication, a kanamycin-resistance marker, and an AIDA-I autotransporter (AT) expression cassette controlled by a rhamnose-inducible promoter. The expression cassette contains a cloning site flanked by BbsI type IIS restriction sites. In its parental configuration, pRAIDA2 expresses an influenza HA immunotag. (**b**) Linear workflow of vaccine production using pRAIDA2 and KWC/GRB (1) antigen identification with codon optimization and structure prediction (AlphaFold or RoseTTAFold); (2) gene synthesis and cloning into pRAIDA2; (3) transformation into genome-reduced bacteria (GRB) for surface display; (4) bacterial growth, expression optimization, and chemical fixation to generate killed whole-cell (KWC) vaccines; (5) immunization of mice for in vivo evaluation. The right-hand arrow denotes the approximate end-to-end timeline (~3 weeks). Abbreviations: AT, autotransporter; GRB, genome-reduced bacteria; KWC, killed whole-cell; HA, hemagglutinin.

We developed a new vaccine platform designed to have many of the most important characteristics desired in a new vaccine platform: Killed Whole-Cell Genome-Reduced Bacteria (KWC/GRB) vaccines that express recombinant immunogens (Imms) on their surfaces by means of a Gram-negative AT [12]. Gram-negative autotransporters (ATs), which enable Gram-negative bacteria to place proteins into the outer membrane [29,30,31,32], have 3 domains: an N-terminal signal sequence that helps mediate transport across the Gram-negative bacterial inner membrane and is subsequently cleaved; a C-terminal β-barrel that inserts into the outer membrane, to yield a structure with a central pore; and a central passenger protein domain that transits through the pore, to place the passenger protein into the extracellular space [33,34]. The autotransporter’s original passenger protein sequence can be replaced by sequence encoding a different protein of interest, like a vaccine Imm, yielding recombinant ATs that display ~2 × 10^5^ passenger proteins on each cell [30,31,35]. There are two types of ATs: a monomeric type and a trimeric type, exemplified by the *H. influenzae* Hia AT [36,37,38]. The trimeric AT structure closely resembles the stem/stalk regions of viruses with Class I viral fusion proteins, like the influenza hemagglutinin, the coronavirus spike protein, or the retrovirus Membrane-Proximal External Region (MPER). The ATs can be used to express multimeric Imms, along with immunomodulators to enhance immune responses [39,40]. We combined the KWC vaccine concept with contemporary synthetic and structural biology approaches to create a synthetic-biology-informed KWC platform that uses ATs to display vaccine antigens on the surfaces of inactivated GRB. Because GRB have many genes encoding surface-exposed proteins deleted, antigen visibility is enhanced [12]. We constructed synthetic plasmids containing a rhamnose-inducible AT expression cassette (Figure 1a) and worked with a gene synthesis company to synthesize DNA encoding the Imms, which were then cloned into the expression vector with an approximately two-week turnaround. As a result, the KWC/GRB platform can yield a testable vaccine in roughly three weeks from Imm selection, enabling the iterative improvements in immunogenicity described here (Figure 1b).

KWC bacterial vaccines, also known as ‘bacterins’, have a long history: currently licensed KWC vaccines against deadly diseases, e.g., cholera, are produced in factories around the world, including in LMICs [26]. KWC vaccines are stable at 2–8 °C for 24 months and cost < $0.1/dose. The UNICEF price for the combination DTP vaccine, including the KWC pertussis component, is $0.25/dose in LMICs33 [41], vs. $14–20/dose for influenza vaccines (per the CDC purchase price list [42]). KWC *E. coli* prophylactic vaccines (e.g., J-Vac, Boehringer Ingelheim), administered IM, have also been widely studied and approved for agricultural use with no problems attributed to lipopolysaccharide (LPS) in the vaccine [43,44,45,46,47,48]. An ideal vaccine platform would combine safety, scalability, low cost, and rapid adaptability to emerging or variant-prone pathogens [49,50]. It should allow for precise antigen presentation and induce both innate and adaptive immune responses. Engineered KWC bacterial vaccines, which have long been used against diseases such as cholera and typhoid [51,52], represent a promising platform for next-generation vaccines.

In our initial use of the platform [12], we made a vaccine targeting the fusion peptide (FP) region of the coronavirus Porcine Epidemic Diarrhea Virus (PEDV) spike protein. In that study, we used a vaccine that included a single copy of the PEDV FP, without any additional immunomodulators, but were nevertheless able to demonstrate substantial clinical protection in pigs after challenge with PEDV, although the induction of anti-PEDV neutralizing antibodies was poor. Given the finding that the single FP vaccine could elicit a protective response, the study suggested that if we could learn how to make an FP vaccine with improved immunogenicity, we could elicit anti-FP neutralizing antibodies and a more protective anti-PEDV immune response.

Due to its low costs and rapid production timeline, the KWC/GRB platform offers the potential to rapidly and iteratively explore strategies to improve immunogenicity of target vaccine Imms. To test the potential power of an immunogenicity improvement campaign, we selected the HIV-1 gp41 fusion FP as a model immunogen. The FP is a short, highly conserved, hydrophobic epitope at the N-terminus of gp41 (≈15–25 amino acids in length) that plays a critical role in virus–cell membrane fusion during entry [53,54,55]. Although the full FP covers a longer region, only ~8 N-terminal residues become exposed during transient conformational changes, and these are the residues accessible to antibody binding [56]. These exposed residues constitute the epitope targeted by broadly neutralizing monoclonal antibodies (bn-mAbs) such as VRC34.01 [57,58]. Structural studies and immunization trials in animals have demonstrated that anti-FP cross-reactive antibodies can be produced [59,60]. The HIV FP has been the subject of vaccine development efforts, but the work has been difficult, due in part to the HIV FP, short length, flexibility, and low. The HIV FP then would present a challenging test case for novel vaccine platforms.

Although the HIV-1 FP is not an ideal antigen for demonstrating neutralizing activity, we selected it intentionally as a technically demanding model for evaluating how design principles, linker architecture, multimerization, and immunomodulators influence antigen display within a DBTL framework [55,61]. Because the FP is short, hydrophobic, and conformationally flexible [54,62], it provides a stringent test of the platform’s ability to improve immunogenicity even when neutralization is not expected. This design choice allowed us to focus on platform optimization rather than protective efficacy, consistent with the goals of a platform-development study [63].

In this study, we present a proof-of-concept Imm improvement campaign using our KWC/GRB vaccine platform with the HIV-1 FP as the target Imm. We designed 20 candidate vaccines, down-selecting after structure prediction to produce 13 representative constructs that explored multiple strategies to enhance FP-directed immunity: single-unit versus multimeric designs, alternative linker architectures, and the incorporation of immunomodulatory sequences including TLR agonists and a non-cognate T-helper epitope. FP surface display was confirmed for all candidates by flow cytometry using the bn-mAb VRC34.01, and immunogenicity was evaluated in HET3 mice by ELISA and pseudovirus-neutralization assays. Across iterative DBTL cycles, we achieved large, design-driven increases in anti-FP antibody magnitude—exceeding an order of magnitude overall—demonstrating the capacity of the KWC/GRB platform to rapidly and substantially enhance immunogenicity in response to rational engineering. However, the sera did not neutralize HIV-1, likely because the isolated FP Imm does not adopt a native-like conformation compatible with the induction of functional neutralizing antibodies.

Overall, our findings support the use of the KWC/GRB vaccine platform as a rapid, inexpensive, easily modified, readily manufactured new vaccine technology, when used with an Imm that has a structure close to the target of broadly neutralizing immune response.

## 2. Materials and Methods

### 2.1. Vaccine Design

We used the four principal known HIV-1 FP sequence variants (FP1–FP4) representing naturally occurring variations within the fusion peptide region of gp41: FP1 (AVGIGAVF), FP2 (AVTIGAVF), FP3 (AVGLGAVF), and FP4 (AVGIGAMF). The FPs were expressed as single FP units, or as multimers (1-, 5-, 8-, or 10-mer) separated by different linker sequences: acidic GDGDG spacers, or a longer linker designed to assume an α-helix structure (AQQASSS × 3). For some designs we added immunomodulatory sequences. These included FLIC (TLR5 agonist fragment) [64,65,66], PADRE (universal CD4^+^ T-helper epitope) [67,68], MASTOPARAN (mast-cell activator) [69,70,71], and rSIP (Group B streptococcal surface immunogenic protein, a TLR-2 and 4 agonist) [17].

### 2.2. D Structure Prediction

After developing each candidate Imm design, we predicted its structure using AlphaFold2 v2.3.0 [72,73]. Models were generated using the monomer_ptm preset with template search enabled, three recycles, and default MSA generation parameters. Amber relaxation was disabled. For each vaccine design, we performed five independent prediction runs using different random seeds, generating five models per run. For multimeric constructs, the repeated FP–linker units were modeled as a single continuous polypeptide. The model with the highest pLDDT and pTM scores was selected for visualization in UCSF ChimeraX [74,75,76]. Predicted structures were used qualitatively to guide design decisions; no quantitative structural restraints were inferred. The AIDA-I AT domain corresponded to residues Q962–F1286 of UniProt entry Q03155.

### 2.3. Plasmid Construction

DNAs encoding the antigens were synthesized, cloned into the rhamnose-inducible pRAIDA2 plasmid, and sequence-verified by Twist Bioscience, San Francisco. pRAIDA2 includes a high-copy origin of replication, a kanamycin resistance marker, and an AIDA-I AT surface expression cassette (Figure 1b; Supplementary File S1).

### 2.4. Bacterial Strain and Transformation

The genome-reduced *Escherichia coli* strain ME5125 (29.7% of the genome deleted), derived from MG1655, was provided by Dr. J. Kato (Tokyo Metropolitan University, Japan) via the National Bioresource Project [77,78]. ME5125 was selected as the chassis for this study for three reasons. First, it ensures continuity with the foundational work by Maeda et al. using the same strain within the KWC/GRB platform framework [12]. Second, ME5125 carries a substantially larger genome reduction (29.7%) than other commonly referenced genome-reduced strains such as MDS42 (~14.3% reduction) or Δlac derivatives (<1% reduction) [79], resulting in a lower background proteome while maintaining robust growth and genetic stability as characterized by Hashimoto et al. and Kato et al. Finally, ME5125 was readily available through the National Bioresource Project, facilitating reproducibility and standardization across studies using genome-reduced bacterial chassis.

Cultures were maintained in LB broth or on LB agar plates at 37 °C, with kanamycin (50 µg/mL) following transformation with the pRAIDA2-derived expression plasmids.

To prepare electrocompetent cells, ME5125 was grown overnight at 37 °C, diluted in fresh LB, and cultured to log phase as determined by serial OD600 measurements. Cells were washed with ice-cold distilled water–10% glycerol, resuspended in water–10% glycerol, and electroporated with the recombinant pRAIDA2 constructs containing the Imm sequence using 0.1 cm cuvettes and a Gene Pulser Xcell (Bio-Rad, Hercules, CA, USA) at 1800 V, 25 µF, 200 Ω. Cells were recovered in SOC medium for 1 h at 37 °C before plating on selective agar.

### 2.5. Colony PCR Verification

To confirm successful transformation, 3–5 kanamycin-resistant colonies per construct were screened by colony PCR. A sterile pipette tip was used to touch each colony, then immediately dipped into 5 mL LB broth with kanamycin to seed the overnight starter culture corresponding to that colony; the same tip was subsequently swirled directly into a 16 µL Phusion Flash High-Fidelity Master Mix reaction (Thermo Fisher Scientific, Waltham, MA, USA) containing primer pair pRAIDA2 forward (5′-CAGCATATGCACATGGAACA-3′) and pRAIDA2 reverse (5′-CATAACTTCCGTTCTCCGGT-3′). PCR cycling parameters were 95 °C for 2 min; 35 cycles of 95 °C for 45 s, 58 °C for 45 s, and 72 °C for 1 min 20 s; and a final extension at 72 °C for 5 min. Amplicons were resolved on 1.5% agarose/TAE gels and visualized under UV illumination; colonies yielding the expected band were selected for subsequent studies. The corresponding 5 µL LB starter culture was expanded overnight to provide the seed inoculum for vaccine-production batches, and an aliquot of the same culture was mixed 1:1 with sterile 30% glycerol (final 15%) and stored at −80 °C as a long-term master stock.

### 2.6. Vaccine Production

Overnight cultures were performed in LB with kanamycin and grown at 37 °C with shaking (210 rpm). The next day, the overnight culture was diluted at OD600 = 0.3 and grown to OD600 = 0.5–0.6, then induced with 5 mM L-rhamnose for 2 h at 37 °C. Bacteria were harvested by centrifugation (5000× *g*, 20 min, 4 °C), resuspended in Hank’s Balanced Salt Solution (HBSS) containing 0.2% formalin, and inactivated at 37 °C for 1 h. After the inactivation step, the bacteria were washed twice with 1× PBS. Final vaccine preparations were resuspended in PBS + 20% glycerol at OD600 = 1.0 (~8 × 10^8^ bacteria/mL), aliquoted, and stored at −80 °C.

### 2.7. Post-Inactivation Testing

Each vaccine lot was tested for sterility immediately after the third PBS wash. Triplicate 100 µL aliquots were serially diluted 10-fold (10^0^–10^−4^) and spread-plated on LB agar (37 °C, 7 days; detection limit 0.2 CFU mL^−1^), while a parallel 1 mL sample was inoculated into 10 mL LB broth and incubated at 37 °C for 14 days; no colony growth or turbidity was observed for any lot, confirming complete inactivation.

### 2.8. Flow Cytometry

Formalin-inactivated bacterial suspensions (5 × 10^7^ cells/mL) were blocked in PBS with 20% fetal bovine serum (FBS) (Corning) for 30 min on ice. After washing with PBS–2% FBS, samples were incubated with 2 µg/mL of anti-HIV FP monoclonal antibody VRC34.01 for 30 min on ice, washed, and then stained with Alexa Fluor 488-conjugated goat anti-human IgG (1:600, Invitrogen, Waltham, MA, USA) for 30 min. Data were acquired on an Attune CytPix flow cytometer (Invitrogen) and analyzed using FCS Express 7. Gating was based on forward and side scatter, and binding percentage was recorded relative to unstained controls. Controls included untransformed ME5125 bacteria processed identically to establish background binding, a PBS blank to monitor instrument background, an isotype antibody to quantify non-specific binding, and a secondary-only condition to measure autofluorescence. Flow cytometry studies were performed at the University of Virginia Flow Cytometry Core.

### 2.9. Mouse Immunization

Quasi-outbred HET3 mice (Jackson Laboratory genetic stock; F1 [CByB6F1 × B6C3F1]) (catalog 036603) were received at 6 weeks, acclimated for ≥7 days under specific-pathogen-free conditions (12 h light/dark cycle; feed and water ad libitum), and block-allocated into fifteen groups comprising the thirteen vaccines plus PBS and formalin-inactivated untransformed ME5125 controls, with 5 mice per group (3 females, 2 males). Animals were immunized intramuscularly in the quadriceps at week 0 (prime), week 3 (boost 1), and week 6 (boost 2) with 5 × 10^9^ inactivated bacteria in 50 µL PBS per dose. Blood was collected by submandibular bleed prior to vaccination (pre-immune, week 0) and at weeks 3, 6, and 9. At week 9, a terminal cardiac exsanguination was performed under deep surgical anesthesia induced by intraperitoneal administration of ketamine (100 mg/kg) and xylazine (10 mg/kg); plasma was isolated (10,000× *g*, 10 min) and stored at −80 °C. Mice were weighed weekly and monitored after each injection for local reactogenicity (erythema, swelling) and systemic signs (ruffled fur, lethargy). No unexpected adverse events were observed.

### 2.10. Enzyme-Linked Immunosorbent Assay (ELISA)

Nickel-coated 96-well plates (Thermo Fisher Scientific, 15442) were loaded overnight at 4 °C with 6× His-tagged recombinant HIV-1 FP peptide (GDDGDGDG-FP; Biomatik, Kitchener, ON, Canada) at 1 µg/mL in coating buffer (PBS, pH 7.4). Plates were washed 3× with TBST (TBS + 0.05% Tween-20) and blocked for 1 h at room temperature with 3% BSA/TBST. Plasma samples were serially diluted 1:50 to 1:102,400 (4-fold series) in 1% BSA/TBST and added in duplicate. Plates were incubated for 1 h at room temperature. Bound IgG was detected with HRP-conjugated goat anti-mouse IgG (Thermo Fisher Scientific; 1:5000) for 1 h, followed by TMB substrate. Reactions were stopped with 1 N H_2_SO_4_ and absorbance read at 450 nm. Each plate included an 8-point VRC34.01 standard curve (starting at 1 µg/mL; 3-fold dilutions) to monitor assay performance and to generate relative antibody unit (RAU) values using a 4-parameter logistic fit. Additional controls included blank wells (no antigen), secondary-only wells, a pre-immune serum pool, and serum from mice immunized with untransformed ME5125.

### 2.11. Neutralization Assays

Neutralization assays were conducted at Duke University using HIV-1 Env pseudoviruses-encoding luciferase and TZM-bl cells [80,81] under standard operating procedures associated with NIAID Contract #75N93025C00006. Briefly, mouse plasma was heat-inactivated (56 °C, 30 min) and serially diluted in infection medium; diluted plasma was incubated with pseudovirus for 1 h at 37 °C and then added to TZM-bl cells. After ~48 h, luciferase activity was measured on a plate reader. Percent neutralization was calculated as 100 × (1 − RLU_{sample}/RLU_{virus-only}). ID_50_ and ID_80_ titers were defined as the reciprocal serum dilutions reducing RLU by 50% or 80% relative to virus-only controls, respectively. Positive (VRC34.01) and negative (naïve serum or irrelevant IgG) controls were included on each plate; samples not reaching 50% inhibition within the tested range were reported as ID_50_ < lowest dilution.

To contextualize the interpretation of non-neutralizing sera, the specific HIV-1 strains included in the pseudovirus panel were: 25710-2.43 (clade C, tier 2), BJOX002000.03.2 (clade B, tier 2), CH119.10 (clade B, tier 2), and MN.3 (clade B, tier 1A). Thus, three of the four pseudoviruses represent difficult-to-neutralize tier 2 strains, providing a stringent assessment of breadth. The fusion peptide (FP; HXB2 positions 512–519) sequences encoded by these pseudoviruses were aligned with the FP variants present in our immunogens. Three pseudoviruses—25710-2.43, BJOX002000.03.2, and CH119.10—encode the FP1 sequence (AVGIGAVF), whereas MN.3 encodes a divergent FP sequence (RAAIGALF) (Table 1 and Table 2). This information is provided to document the FP sequence and tier context relevant to the neutralization assays.

### 2.12. Statistical Analysis

All statistical analyses were performed in R (v4.4.0) using the tidyverse, ggplot2, readxl, writexl, stringr, drc, and base stats packages within the RStudio (2023.12.1+402) integrated development environment. The analytical workflow, including all scripts for curve fitting, data processing, and hypothesis testing, was developed to ensure reproducibility, internal consistency, and transparent documentation. All raw datasets and statistical scripts are provided in Supplementary Files S2 and S3.

Quantitative endpoints derived from ELISA and flow cytometry included:

(1) exposure_score = −log_10_(EC_50__FLOW), reflecting antigen surface accessibility;
(2) AUC_FLOW, representing the integrated magnitude of antibody binding in flow cytometry;
(3) AUC_ELISA, used as the primary measure of circulating humoral response; and
(4) Bmax_ELISA, summarizing maximal binding signal.

Endpoints were obtained by fitting raw data to a four-parameter logistic (4PL) model under uniform bounds. Curves lacking sufficient dynamic range were excluded or flagged as NA.

The statistical workflow proceeded through several analytical layers. First, raw curves were fitted, and quantitative parameters were extracted as described above. Second, relationships among exposure_score, AUC_FLOW, and AUC_ELISA were examined using Spearman’s rank correlation (ρ), while Kendall’s τ was used to assess temporal trends across design iterations. Third, differences across multi-group design variables (Composition, Linker, Immunomodulators, Conformation) were evaluated using non-parametric Kruskal–Wallis tests. When justified, Mann–Whitney U or Wilcoxon rank-sum tests were used for two-group comparisons, and Hodges–Lehmann effect estimates with 95% confidence intervals were reported. Fourth, to isolate the effect of individual design elements (e.g., PADRE or FLIC), paired Wilcoxon signed-rank tests were applied to Δ(Hi–Lo) contrasts between vaccine constructs differing only in the factor of interest, with structured pairing rules and predefined relaxations when perfect matching was not feasible. Fifth, analyses were repeated at the individual animal level using Mann–Whitney U tests on AUC_ELISA values to validate vaccine-level findings. Finally, a multivariate Principal Component Analysis (PCA) was performed on scaled immunogenicity variables (AUC_ELISA, exposure_score, AUC_FLOW) to generate a composite index (PC1) integrating antigen exposure and antibody magnitude.

To ensure appropriate control of Type I error in multi-group analyses, assumptions of normality and variance homogeneity were evaluated prior to selecting statistical procedures. Because ELISA- and flow-derived endpoints did not satisfy parametric assumptions due to small sample sizes and non-normal distributions, classical one-way ANOVA was not applied. Instead, Kruskal–Wallis tests were followed by Dunn’s post hoc test with Benjamini–Hochberg false discovery rate (FDR) adjustment, providing a rigorous non-parametric framework for comparing EC_50_-derived and AUC_ELISA values across multiple vaccine designs.

All statistical tests were two-sided. Given the exploratory scope and inherent limitations of mouse-sized cohorts, *p*-values were interpreted alongside effect sizes and consistency across analytical layers. A Δ exposure_score ≥ 0.30 (approximately a two-fold improvement corresponding to a halved EC_50_) was predefined as biologically meaningful.

### 2.13. Ethics, Animal Welfare, and Biosafety

All animal procedures were conducted in accordance with the Guide for the Care and Use of Laboratory Animals and were approved by the University of Virginia Animal Care and Use Committee (protocol 41101224; approval date: 5 November 2024; Unique ID: 7018391). Recombinant DNA work and handling of formalin-inactivated *E. coli* vaccine lots were performed under BSL-2 conditions approved by the UVA Institutional Biosafety Committee (IBC protocol 4276-15). The study complied with ARRIVE reporting guidelines where applicable.

## 3. Results

### 3.1. Iterative Design–Build–Test–Learn (DBTL) Workflow and Candidate Curation

**Figure 2:**
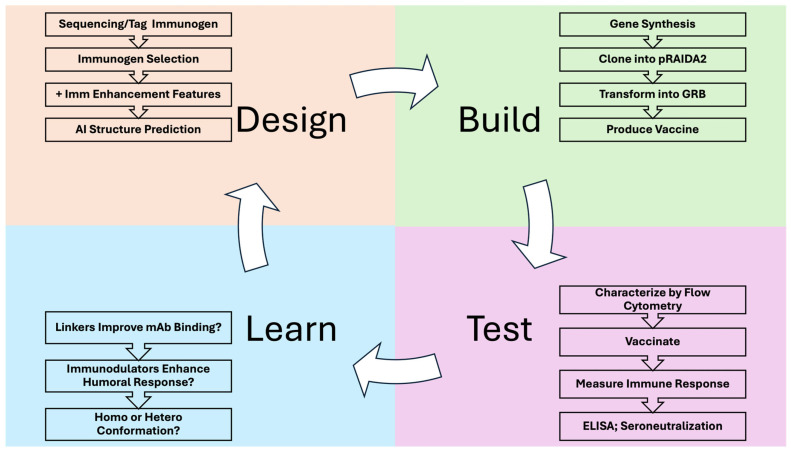
Schematic overview of DBTL cycle used to iteratively optimize vaccines produced with the KWC/GRB platform. Abbreviations: DBTL, Design–Build–Test–Learn; KWC/GRB, killed whole-cell/genome-reduced bacteria.

To evaluate our killed whole-cell bacterial vaccine technology, after initial work with PEDV fusion peptide (FP) [12] we selected the HIV-1 (FP)—a highly conserved, hydrophobic region of gp41—as a model antigen. The FPs of many viral envelope proteins (e.g., coronavirus stalk, influenza HA, HIV Env) are the target of well-characterized bn-mAbs and have been explored previously as vaccine targets [53,54,55,56,57]. We applied a DBTL cycle to generate ~30 FP-based vaccines varying FP1 repeat number, linker architecture (L1, L2, L3), conformational context, and immunomodulatory domains (Figure 2). In Stage 1 (design), candidates were screened in silico with AlphaFold2; designs predicted to compromise epitope display were not advanced. Our prototypical comparator was a 1mer FP bearing an N-terminal 3DA motif (DADADA) shown to facilitate AIDA-I export (see below). This 1mer configuration parallels the single-FP coronavirus design from our initial KWC/GRB report [12], which protected pigs against PEDV challenge but did not elicit robust neutralizing antibodies. Sixteen designs were screened out at this stage. In Stage 2 (build & expression), DNA was synthesized, cloned into the AIDA-I expression cassette (pRAIDA2) (Supplementary File S1), transformed into genome-reduced *E. coli* ME5125, and surface expression was assessed by flow cytometry using the bn-mAb VRC34.01; binding percentage was used as the readout. In Stage 3 (test & learn), down-selected vaccines advanced to mouse immunization and serology (ELISA); results informed the next design round. For example, “5mer GGG” showed acceptable AlphaFold2 structure but no binding percentage gain versus 1mer and was not taken forward.

### 3.2. Optimization of Surface Antigen Exposure Using the 3DA Motif

**Figure 3:**
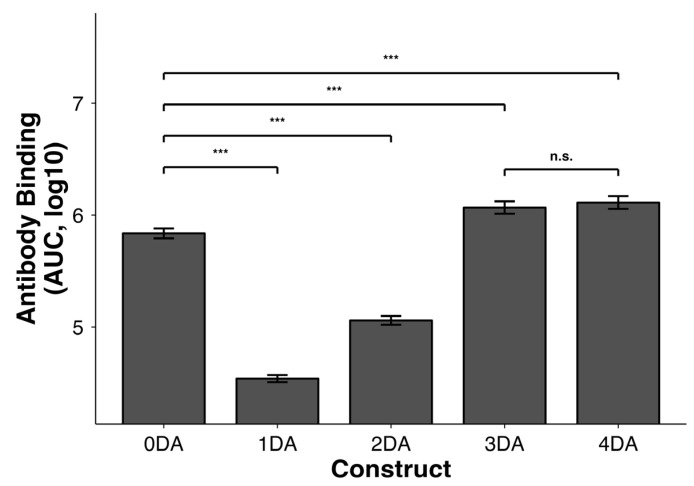
Flow cytometry quantification of COV44-79 binding to KWC/GRB expressing a 1mer SARS-CoV-2 FP with N-terminal DA repeats. Bars show mean ± SD of AUC (log_10_) for 0–4DA (n = 5 per group). All DA variants were statistically different from 0DA (Welch’s *t*-test, *p* < 0.001), but only 3DA and 4DA showed a significant increase in antibody binding. The top-performing variants (3DA and 4DA) were not significantly different from each other (n.s.). Abbreviations: KWC/GRB, killed whole-cell/genome-reduced bacteria; FP, fusion peptide; DA, aspartic acid; AUC, area under the curve; SD, standard deviation; n.s., not significant. *** *p* < 0.001.

Previous work by Maeda et al. demonstrated that immunization with KWC/GRB expressing a 1mer SARS-CoV-2 fusion peptide conferred protection in pigs, despite the absence of detectable antibodies by ELISA. This finding suggested that protection might involve non-humoral mechanisms and that if we were able to make a vaccine that induced a good humoral immune response we might be able to see even better protection. We hypothesized that we would be able to induce better antibody responses if we used a multimeric version of the antigen, because a multimeric antigen would provide an enhanced Imm dose to the animals and because a multimeric Imm might enable more avid binding to B-cell precursors, since the overall binding constant would be the product of multiple lower affinity constants. A multimeric Imm may also initiate multiple signal transduction events to further enable selection of B-cell precursors with initial low affinity B-cell receptors for subsequent affinity maturation. To enhance Imm recognition on the bacterial surface, we conducted optimization experiments using multimers of the same antigen, introducing 1 Asp-Ala (DA), and 2DA, 3DA, and 4DA motifs at the N-terminal side. Here we aimed to make the N-terminal region of the expressed Imm more hydrophilic to help improve the thermodynamic drive of the AT-mediated export process (export of the AT native or recombinant passenger protein does not require high energy phosphate and instead is driven by the free energy of solvation of the passenger protein in the extracellular medium). We also aimed to provide the expressed highly hydrophobic FPs with a greater net negative charge in an attempt to minimize its interactions with the outer membrane lipid bilayer. Flow cytometry with the monoclonal antibody COV44-79 [82] revealed that vaccines containing the 3DA or 4DA motifs exhibited the highest levels of antibody binding (AUC = 6.07 ± 0.06 and 6.11 ± 0.06, respectively). Both configurations showed significantly greater binding than 0DA, 1DA, and 2DA (*p* < 0.001), while the difference between 3DA and 4DA was not statistically significant (*p* = 0.24), as determined by Welch’s *t*-test (Figure 3). These results indicate that incorporation of three DA repeats provides an optimal balance between antigen exposure and structural economy. Based on this finding, all HIV-FP vaccines described here were engineered to include the 3DA motif to maximize surface antigen accessibility and antibody detectability.

### 3.3. A Campaign to Improve Immunogenicity Using HIV FP

A total of 13 vaccine candidates showed VRC34.01 binding equal to or better than that shown by the vaccine that expressed the 1-mer FP in flow cytometry experiments. These vaccines had several features designed to enhance the antibody response induced by the vaccines. These features included multimeric versions of different variants of the FP Imm, with added immunomodulators, including TLR agonists and T-cell antigens. Table 3 outlines the structural and immunological roles of the protein domains included in the candidate vaccines. Table 4 presents an overview of the vaccine constructs organized by design rounds, including their names, abbreviations, and composition. Table 3 and Table 4 summarize domain functions and vaccine compositions by design round.

Each design round incorporated progressively refined strategies to improve antigen display, enhance immunogenicity, and evaluate the modular capacity of the platform. This iterative process served as a real-time “test–learn–optimize” cycle for platform refinement.

### 3.4. First-Round Vaccines: Minimalist Designs and Baseline Performance

**Figure 4:**
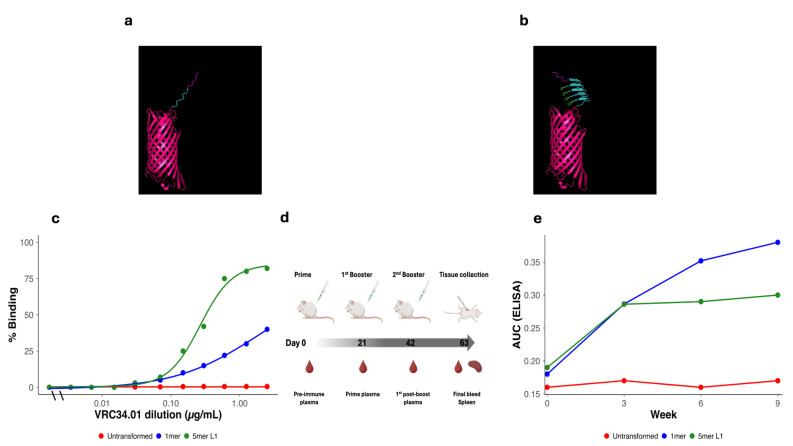
Structural design, surface expression, and immunogenicity of first-round of HIV-FP vaccines. (**a**) Predicted structural of 1mer displayed 3DA motif (magenta), FP1 (cyan), and AIDA-I β-barrel (pink). (**b**) 3D structure of 5mer L1. The 3DA motif (magenta) is positioned at the N-terminus, followed by five tandem FP1 repeats (cyan). These are separated by GDGDG linker (L1, green), extending outward from the AIDA-I β-barrel domain (pink). (**c**) Flow cytometry of *E. coli* ME5125 expressing untransformed (red), 1mer (blue), and 5mer L1 (forest green) stained with VRC34.01; the 5mer L1 showed higher binding percentage. (**d**) Immunization schedule in HET3 mice with inactivated KWC/GRB HIV-FP candidates at weeks 0, 3, and 6; bleeds at weeks 0, 3, 6, and 9. (**e**) AUC_ELISA at weeks 0, 3, 6, and 9 for Untransformed (red), 1mer (blue), and 5mer L1 (forest green). Lines and points show the mean AUC across animals per time point, illustrating the longitudinal increase in antibody magnitude with multimerization. Abbreviations: FP, fusion peptide; FP1, HIV-1 fusion peptide variant 1; 3DA, three aspartic acid residues; L1, GDGDG linker; 1mer/5mer, number of tandem FP repeats; AIDA-I, autotransporter domain; KWC/GRB, killed whole-cell/genome-reduced bacteria; HET3, quasi-outbred mouse stock; AUC_ELISA, area under the ELISA curve.

Initial vaccines were designed to evaluate the basic functionality of the KWC/GRB platform using FP antigens alone. These included a single-copy FP vaccine (1mer) and a multimeric five-copy vaccine (5mer) design with the FP units separated by GDGDG linkers (L1). For these initial studies, we used the most common FP sequence, which we term FP1 (Table 3). AlphaFold2 structure prediction suggested that the AIDA-I AT β-barrel domain would be properly folded, and that the FP repeats would extend away from the β-barrel (Figure 4a,b).

Flow cytometry confirmed VRC34.01 binding to surface-displayed FP on KWC/GRB. As expected for multimerization, 5mer vaccine increased binding percentage, although the gain was not proportional to repeat number (Figure 4c). One potential concern was that the hydrophobic FP domains were predicted to stack up on top of each other, making some of the FP units less accessible to the VRC34.01 mAb than might have been desired.

We vaccinated HET3 mice intramuscularly with the KWC/GRB constructs (Figure 4d). ELISAs on serial bleeds showed low yet detectable anti-FP titers by week 9 (Figure 4e). No neutralization was detected against a panel of HIV-1 pseudoviruses in TZM-bl assays, including three tier-2 viruses whose FP sequences are identical to FP1. To document the structural context of these findings, we summarized the predicted AlphaFold pLDDT values for the FP region together with antigen-exposure (AUC_FLOW), antibody-response magnitude (AUC_ELISA), and neutralization outcomes in Supplementary File S2—Table S4. Across constructs, pLDDT values were uniformly low (<70, frequently <50), consistent with limited predicted structural confidence in the FP region under these display configurations.

To establish a baseline for interpreting vaccine-induced improvements, we quantified antigen exposure and antibody responses in untransformed ME5125 controls. As expected for bacteria lacking FP expression, untransformed ME5125 showed minimal surface reactivity in flow cytometry (AUC_FLOW = 1.26) and negligible ELISA signal (AUC_ELISA ≈ 0.01). In contrast, both the 1mer construct and the multimeric 5mer designs displayed substantially higher antigen exposure and antibody magnitudes, confirming that the increases observed across subsequent design rounds represent true immunogenic gains attributable to vaccine engineering rather than assay background.

### 3.5. Second-Round Vaccines: Enhancing Display and Adding Immunomodulators

To overcome the low immunogenicity observed with first-round vaccines, the second-round vaccines incorporated several modifications aimed at improving antigen exposure and anti-FP response. These included: (i) the use of L2 (AQQASSS) × 3 rigid α-helical linkers to separate the FP Imm units away from each other. We hypothesized additionally that the L2 linker would further increase inter-FP spacing to support multivalent B-cell engagement and potential avidity gains, and to further project the expressed antigen out from the bacterial outer membrane; (ii) the increase in FP Imm copy number to five or ten repeats in an effort to increase Imm dose per bacterium and to attempt to further increase avidity between the KWC/GRB vaccine and the B-cell precursors; and (iii) the addition of immunomodulatory domains, including PADRE (a non-cognate universal pan-DR T helper epitope), FLIC (a flagellin-derived TLR5 agonist), and rSIP (a TLR2/4 agonist from Group A Streptococcus) (see Table 3 for details of each domain and Table 4 for vaccine compositions).

**Figure 5:**
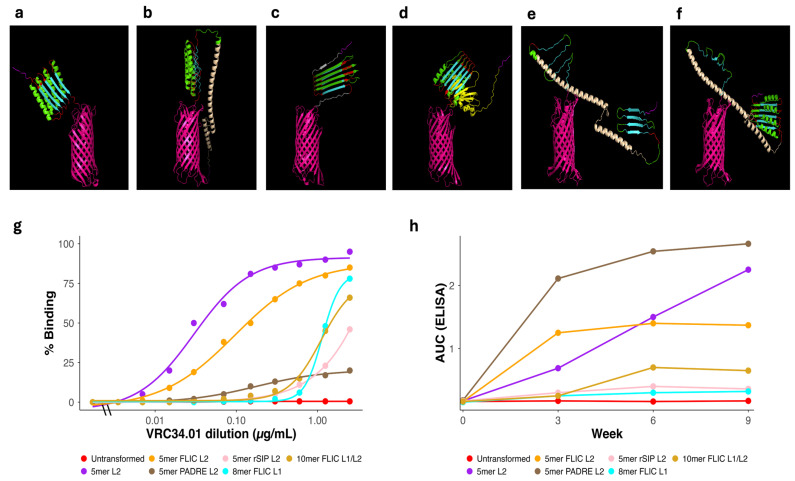
Structural design, surface expression, and immunogenicity of second-round of HIV-FP vaccines. (**a**) Predicted structure of the 5mer L2 vaccine. The 3DA motif (magenta) is positioned at the N-terminus, followed by five tandem FP1 repeats (cyan). These are separated by an α-helical linker (L2, green) and a short flexible linker (L3, red), extending outward from the AIDA-I β-barrel domain (pink). (**b**) Predicted structure of the 5mer-FLIC L2 vaccine, containing the 3DA motif (magenta) and FLIC (wheat) at the N-terminus. These are followed by five FP1 repeats (cyan), separated by L2 (green) and L3 (red) linkers, and anchored by the AIDA-I β-barrel domain (pink). (**c**) Structure of the 5mer-PADRE L2 vaccine, in which the 3DA motif (magenta) is fused to PADRE (white). The design preserves the arrangement of five FP1 repeats (cyan) and the L2 (green) and L3 (red) linkers, mirroring the organization described in panel A. (**d**) Structure of the 5mer-rSIP L2 vaccine, in which the 3DA motif (magenta) is fused to rSIP (yellow). This vaccine maintains the same organization of FP1 repeats (cyan), L2 (green), and L3 (red) linkers as shown in panel A. (**e**) Predicted 3D structure of the 8mer-FLIC L1 vaccine. The 3DA motif (magenta) precedes four FP1 repeats (cyan), separated by L1 linkers (green), followed by FLIC (wheat), and then another set of four FP1 repeats (cyan), also separated by L1 linkers (green). (**f**) Predicted 3D structure of the 10mer-FLIC L1/L2 vaccine. The 3DA motif (magenta) is followed by five FP1 repeats (cyan), separated by L1 (green), then by FLIC (wheat), and finally by an additional set of five FP1 repeats (cyan), L2 (green), and L3 (red) linkers. (**g**) Flow cytometry analysis of *E. coli* ME5125 expressing untransformed (red), 5mer L2 (purple), 5mer-FLIC L2 (orange), 5mer-PADRE L2 (clay brown), 5mer-rSIP L2 (pink), 8mer FLIC L1 (cyan), and 10mer FLIC L1/L2 (goldenrod yellow) vaccines stained with the broadly neutralizing antibody VRC34.01. Vaccines lacking immunomodulators or containing FLIC exhibited increased binding percentage of the FP epitope with VRC34.01. (**h**) AUC_ELISA at weeks 0, 3, 6, and 9 for Untransformed (red), 5mer L2 (purple), 5mer FLIC L2 (orange), 5mer PADRE L2 (clay brown), 5mer rSIP L2 (pink), 8mer FLIC L1 (cyan), and 10mer FLIC L1/L2 (goldenrod yellow). Lines and points indicate the temporal evolution of humoral magnitude; vaccines carrying the extended L2 linker and PADRE/FLIC show the largest increases over time. Abbreviations: FP1, fusion peptide variant 1; 3DA, three aspartic acid residues; L1/L2/L3, linker variants; FLIC, flagellin-derived immunostimulatory peptide; PADRE, universal T-helper epitope; rSIP, synthetic immunomodulatory peptide; 1mer/5mer/8mer/10mer, number of FP repeats; AIDA-I, autotransporter domain; KWC/GRB, killed whole-cell/genome-reduced bacteria; AUC_ELISA, area under the ELISA curve.

Structural prediction of these designs confirmed the desired folding and spatial separation of the antigenic domains and linkers, with preservation of immunomodulator architecture (Figure 5a–f). These predictions indicated that the longer α-helical linkers would likely be successful in separating the FP Imms from each and in distancing the FP from the bacterial outer membrane.

Flow cytometry analysis revealed that the broadly neutralizing antibody VRC34.01 bound most efficiently to vaccines incorporating L2 consistently showed higher binding percentage; FLIC-containing design was among the top binders (Figure 5g).

ELISA analyses of serial bleeds demonstrated markedly higher anti-FP antibody titers in mice immunized with 5mer vaccines containing L2 linker and PADRE or FLIC (Figure 5h). Vaccines incorporating these immunomodulators generated the highest AUC_ELISA values, showing substantially stronger responses than the minimal FP constructs from the first design round. These findings highlight the critical contribution of both structural optimization and immunomodulatory design elements in maximizing humoral responses within the KWC/GRB vaccine platform.

Despite the increased ELISA responses observed in this design round, neutralization assays performed in TZM-bl cells using a panel of HIV-1 pseudoviruses detected no measurable neutralizing activity for any of the vaccines. To contextualize these findings, the predicted AlphaFold pLDDT values for the FP region and the corresponding antigen-exposure and antibody-response metrics for all constructs are summarized in Supplementary File S2—Table S4. Across constructs, the predicted structural confidence of the FP region remained uniformly low (<70, frequently < 50).

### 3.6. Third-Round Vaccines: Enhanced Immuno-Stimulation

The second-round vaccines incorporated extended designs containing up to eight or ten FP1 repeats; however, these failed to produce further improvements in binding signal in flow cytometry assays or in antibody titers following immunization, relative to the 5mer vaccines (Figure 5g,h). These findings prioritized the 5mer backbone for subsequent optimization.

In the next set of experiments, instead of a single uniform FP, we included four FPs that represent some of the known heterogeneity observed among FP sequences [54]. These designs incorporated heteromeric FP sequences (FP1–FP4, Table 3) arrayed in tandem, separated by L2 (AQQASSS) × 3 and the N-terminal 3DA motif (DADADA). We added immunomodulators, FLIC, PADRE, and the mast-cell activator mastoparan, to the FPs (see Table 3 and Table 4).

**Figure 6:**
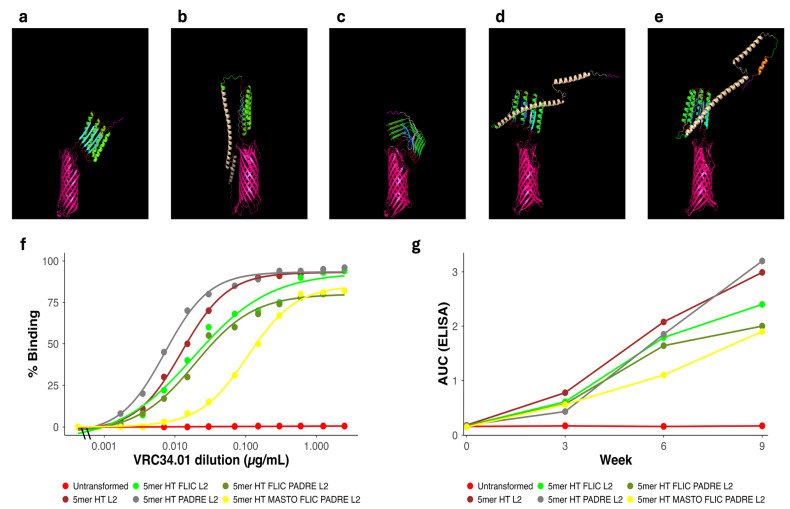
Structural design, surface expression, and immunogenicity of the third-round of HIV-FP vaccines. (**a**) Predicted structure of the 5mer-HT L2 vaccine. The 3DA motif (magenta) precedes the heteromeric FP array FP1–FP2–FP3–FP4–FP1 (shades of blue), separated by α-helical L2 linkers (green) and short flexible L3 linkers (red), and anchored to the AIDA-I autotransporter β-barrel (pink). (**b**) Predicted three-dimensional structure of the 5mer-HT-FLIC L2 vaccine, containing the 3DA motif (magenta) and the FLIC immunomodulator (wheat) at the N-terminus, followed by the FP1–FP2–FP3–FP4–FP1 array (shades of blue), L2 (green) and L3 (red) linkers, and the AIDA-I autotransporter β-barrel (pink). (**c**) Structure of the 5mer-HT-PADRE L2 vaccine, in which the 3DA motif (magenta) is fused to PADRE (white), maintaining the arrangement of FP1–FP2–FP3–FP4–FP1 (shades of blue), L2 (green), and L3 (red) linkers, similar to panel A. (**d**) Structure of the 5mer-HT-FLIC-PADRE L2 vaccine, containing 3DA (magenta) fused to FLIC (wheat) and PADRE (white), preserving the organization of FP1–FP2–FP3–FP4–FP1 (shades of blue), L2 (green), and L3 (red) linkers, as in panel A. (**e**) Structure of the 5mer-HT-MASTO-FLIC-PADRE L2 vaccine, in which the 3DA motif (magenta) is fused sequentially to mastoparan (orange), FLIC (wheat), and PADRE (white), maintaining the FP1–FP2–FP3–FP4–FP1 arrangement (shades of blue) with L2 (green) and L3 (red) linkers, as in panel A. (**f**) Flow cytometry analysis of *E. coli* ME5125 expressing untransformed (red), 5mer-HT L2 (brown), 5mer-HT-FLIC L2 (bright green), 5mer-HT-PADRE L2 (gray), 5mer-HT-FLIC-PADRE L2 (olive), and 5mer-HT-MASTO-FLIC-PADRE L2 (neon yellow) vaccines stained with VRC34.01. Vaccines lacking immunomodulators or containing PADRE and/or FLIC showed higher binding percentage. (**g**) AUC_ELISA at weeks 0, 3, 6, and 9 for Untransformed (red), 5mer HT L2 (brown), 5mer HT FLIC L2 (bright green), 5mer HT PADRE L2 (gray), 5mer HT FLIC PADRE L2 (olive), and 5mer HT MASTO FLIC PADRE L2 (neon yellow). Lines and points summarize longitudinal antibody magnitude across heteromeric designs, highlighting the impact of linker architecture and immunomodulator inclusion. Abbreviations: HT, heteromeric array; FP1–FP4, fusion peptide variants; 3DA, three aspartic acid residues; L2/L3, linker variants; FLIC, flagellin-derived immunostimulatory peptide; PADRE, universal T-helper epitope; MASTO, mastoparan peptide; AIDA-I, autotransporter domain; AUC_ELISA, area under the ELISA curve; KWC/GRB, killed whole-cell/genome-reduced bacteria.

Structure prediction confirmed that these designs had the desired conformations (Figure 6a–e). Flow cytometry with the monoclonal antibody VRC34.01 showed that the highest levels of surface FP exposure were achieved by multimeric constructs incorporating PADRE and/or FLIC, with the 5mer HT PADRE L2 design exhibiting the strongest overall mAb binding among the Round 3 candidates (Figure 6f).

Following vaccination of HET3 mice, the strongest anti-FP antibody responses across the entire campaign were elicited by vaccines featuring heteromeric (HT) conformations (Figure 6g). Constructs incorporating PADRE and/or FLIC consistently induced higher antibody titers, with the 5mer HT PADRE L2 design achieving the greatest overall AUC_ELISA values. The addition of mastoparan further enhanced responses in selected formulations. Together, these findings illustrate the modular capacity of the KWC/GRB platform to integrate antigen-presentation features with immunostimulatory domains to progressively enhance immunogenicity across iterative DBTL rounds.

Despite the marked improvements in FP Imm surface display observed by flow cytometry and the increased anti-FP antibody responses, the sera did not neutralize HIV-1 pseudoviruses in TZM-bl inhibition assays. To document the structural context for these findings, the predicted AlphaFold pLDDT values for the FP region, together with antigen-exposure and antibody-response metrics for all constructs, are summarized in Supplementary File S2—Table S4. Across constructs, the predicted structural confidence of the FP region remained uniformly low (<70, frequently < 50). This is consistent with literature showing that, while FP is a known target of bn-mAbs, isolated FP immunogens rarely elicit neutralization without additional structural or contextual optimization.

### 3.7. Curve-Derived Metrics from Raw Assays (Tabulated Summary)

The curve-derived metrics—EC_50__log10_ELISA (potency), Bmax_ELISA and AUC_ELISA (magnitude), EC_50__log10_FLOW, exposure_score = −log10(EC_50__FLOW), and AUC_FLOW—are summarized in Table 5. Metrics were obtained by 4-parameter logistic (4PL) fits under identical bounds across vaccines; curves with insufficient dynamic range were flagged and set to NA (retained for pairwise-complete analyses). Values are listed in the original vaccine order to preserve the design timeline; these standardized per-vaccine metrics formed the basis for correlation and factor-wise analyses.

### 3.8. Comparison of Correlation Models for VRC34.01 Binding and Antibody Response

In using the KWC/GRB platform to produce new candidate vaccines, it would be useful to be able to select which candidates would be most likely to induce an excellent immune response prior to testing the candidate vaccines in vivo, to minimize animal use and expense and speed the development of new vaccines. In inspecting the data, it appeared likely that a good ability to bind the relevant monoclonal antibody directed against the vaccine Imm would correlate with an ability to induce antibody production. To determine whether the extent of monoclonal antibody binding to the HIV-FP epitope predicts the magnitude of the humoral response elicited in vivo, we compared quantitative binding parameters derived from flow cytometry with antibody titers measured by ELISA across multiple time points. The flow cytometry variable was expressed as the exposure score = −log_10_(EC_50__FLOW) (higher values indicate stronger VRC34.01 binding), while antibody responses were represented as AUC_ELISA.

**Figure 7:**
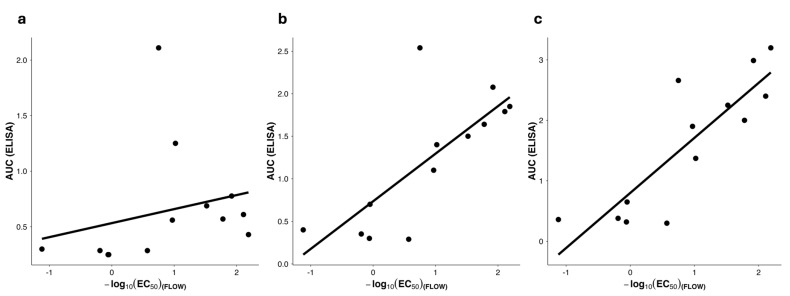
Correlation between VRC34.01 binding and humoral response over time. Correlation between AUC_ELISA and the exposure score = −log_10_(EC_50__FLOW) across vaccines at (**a**) week 3, (**b**) week 6, and (**c**) week 9. Each point represents a distinct vaccine; solid lines indicate linear regression fits. Vaccines exhibiting stronger binding to the neutralizing antibody VRC34.01 (higher −log_10_ EC_50_ values) also elicited higher antibody responses in ELISA, with the correlation strengthening as immunization progressed. Coefficient of determination (R^2^, Pearson) by timepoint: Week 3: R^2^ = 0.16; Week 6: R^2^ = 0.56; Week 9: R^2^ = 0.72. This supports VRC34.01 binding as a quantitative design-gate predictor of immunogenic performance in the KWC/GRB platform. Abbreviations: AUC_ELISA, area under the ELISA curve; EC_50__FLOW, half-maximal effective concentration in flow cytometry; KWC/GRB, killed whole-cell/genome-reduced bacteria.

When integrating data from weeks 3, 6, and 9 (Figure 7), the correlation between VRC34.01 binding and antibody magnitude strengthened over time (Spearman ρ = 0.56, *p* = 0.047 at week 3; ρ = 0.75, *p* = 0.003 at week 6; ρ = 0.82, *p* = 5 × 10^−4^ at week 9). The global correlation across all time points remained highly significant (ρ = 0.65, *p* < 10^−4^). These results indicate that vaccines exhibiting stronger VRC34.01 binding in flow cytometry tend to elicit progressively higher antibody responses as the animals are repeatedly exposed to the vaccine antigens, likely representing continued antibody affinity maturation, particularly as the mice were exposed to multiple vaccine doses. This finding validates VRC34.01 binding as an early quantitative design-gate predictor of vaccine immunogenicity within the KWC/GRB platform.

### 3.9. Cross-Assay Concordance of Integrated Readouts (AUC_ELISA vs. AUC_FLOW)

**Figure 8:**
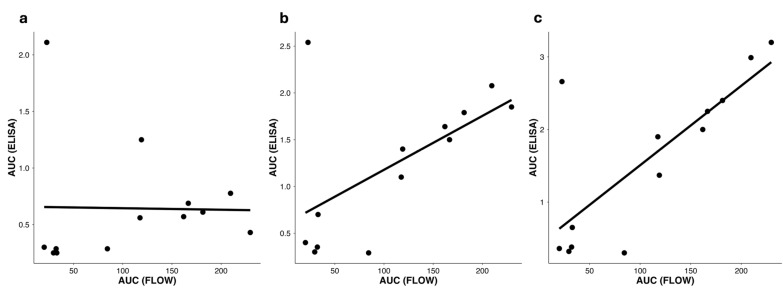
Concordance between integrated ELISA and flow cytometry readouts. Correlation between AUC_ELISA and AUC_FLOW across vaccines at (**a**) week 3, (**b**) week 6, and (**c**) week 9. Each point represents a distinct vaccine, and solid lines indicate linear regression fits. Vaccines with higher antigen exposure on the bacterial surface (larger AUC_FLOW) also exhibited stronger serum antibody responses (higher AUC_ELISA), with the association becoming progressively stronger from week 6 to week 9. Coefficient of determination (R^2^, Pearson) by timepoint: Week 3: R^2^ = 0.34; Week 6: R^2^ = 0.49; Week 9: R^2^ = 0.59. This temporal reinforcement demonstrates that both assays capture a consistent biological signal linking antigen display with the magnitude of the humoral response. Abbreviations: AUC_ELISA, area under the ELISA curve; AUC_FLOW, area under the flow–cytometry binding curve.

To evaluate the agreement between independent quantitative assays, we compared the integrated readouts from ELISA and flow cytometry at weeks 3, 6, and 9 (Figure 8). A significant positive correlation was observed globally (Spearman ρ = 0.47, *p* = 0.0028), becoming progressively stronger at later time points (ρ = 0.36 at week 3, ρ = 0.54 at week 6, ρ = 0.68 at week 9). This concordance indicates that vaccines exhibiting higher antigen exposure on the bacterial surface (Flow Cytometry AUC) also elicit stronger serum antibody responses (ELISA AUC). The temporal progression of this relationship supports the view that antigen display level contributes directly to the magnitude and maturation of the humoral response, further validating both assays as consistent and biologically meaningful readouts for vaccine ranking within the KWC/GRB platform.

### 3.10. Non-Parametric Assessment of Individual Design Factors

Non-parametric analyses were performed to evaluate the contribution of key vaccine design factors—Composition, Linker, Immunomodulators, and Conformation—to antigen exposure (AUC_FLOW, exposure_score = −log_10_EC_50__FLOW) and humoral magnitude (AUC_ELISA). Across pooled comparisons, Hodges–Lehmann estimators with 95% confidence intervals revealed no statistically significant differences among factor levels (all *p* > 0.05), and label-permutation as well as timeline (Kendall’s τ) tests produced concordant null results. These findings reflected the small number of vaccines per category and the high structural heterogeneity among designs.

**Figure 9:**
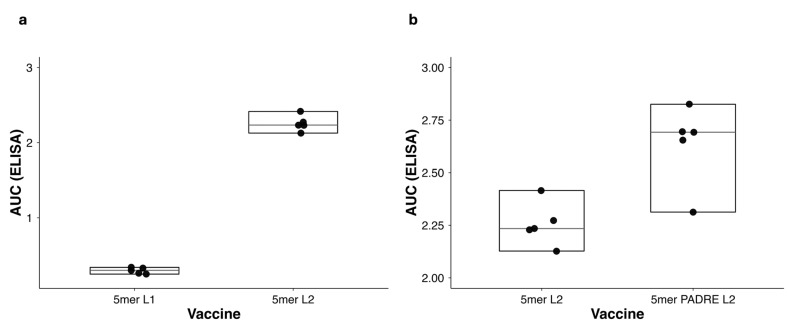
Influence of linker architecture and PADRE inclusion on antibody magnitude. (**a**) Comparison of α-helical linker (L2, AQQASSS × 3) versus short acidic linker (L1, GDGDG) showing higher antibody magnitude for vaccines containing the extended L2 linker (Mann–Whitney U, *p* = 0.0122). (**b**) PADRE inclusion significantly increased the humoral response in HM vaccines carrying the L2 linker compared with their PADRE-negative counterparts (Mann–Whitney U, *p* = 0.0159). Each dot represents the AUC_ELISA value of an individual mouse; boxplots indicate interquartile range, median, and distribution of values. All analyses were two-sided, performed in R (v4.4.0) using non-parametric tests. n = 5 mice per group. Abbreviations: L1/L2, linker variants; PADRE, universal T-helper epitope; HM, homomeric vaccine design; AUC_ELISA, area under the ELISA curve.

When examined separately, Composition (1mer–10mer) showed no consistent association with either antigen exposure or antibody response. Linker architecture demonstrated a clearer trend: vaccines incorporating the longer α-helical linker L2 (AQQASSS × 3) consistently exhibited higher AUC_ELISA values than those containing the shorter acidic linker L1 (GDGDG). Although this effect was not significant in pooled vaccine-level analyses, it became statistically significant when evaluated using individual-mouse AUC_ELISA values (*p* = 0.0122, Mann–Whitney test; Figure 9a), thereby providing a direct pairwise statistical comparison supporting the superior immunogenic performance of the L2 linker.

Paired contrasts between otherwise identical vaccines differing only in the presence of PADRE or FLIC were next evaluated using the Wilcoxon signed-rank test (ΔHi–Lo). Across all available pairs, no statistically significant effects were detected (n = 1–2 per factor). However, these exploratory comparisons revealed a reproducible directional trend toward increased AUC_ELISA and AUC_FLOW values in PADRE-containing formulations, consistent across both homomeric (HM) and heteromeric (HT) contexts.

To better delineate these trends, individual-level analyses were conducted using Mann–Whitney U tests on per-mouse AUC_ELISA values. This approach verified that PADRE significantly increased antibody magnitude within HM vaccines (*p* = 0.0159, Figure 9b), whereas no discernible effect was observed in HT vaccines (*p* > 0.2). FLIC did not produce a statistically significant impact on antibody magnitude in either setting. In parallel, conformation (HM vs. HT) comparisons showed a positive, non-significant trend toward higher antibody levels in HT vaccines (*p* = 0.0947), implying a possible influence of antigen sequence heterogeneity on humoral magnitude.

Finally, an integrated multivariate analysis was performed to visualize global vaccine behavior across quantitative endpoints. Principal Component Analysis (PCA) integrating AUC_ELISA, AUC_FLOW, and exposure_score identified PC1 as the dominant axis capturing the shared variance between in vitro antigen exposure and in vivo antibody response, with PC2 representing secondary structural variability among vaccines. Vaccines with higher PC1 scores corresponded to those exhibiting strong exposure and antibody magnitude, confirming the coherence between surface display and humoral induction previously shown by Spearman correlations (Section 3.8 and Section 3.9).

Collectively, these analyses demonstrate that while most structural parameters showed limited statistical separation at the vaccine level, refined non-parametric and multivariate analyses revealed that linker structure (L2) and PADRE inclusion within HM vaccines are associated with enhanced humoral magnitude, whereas FLIC exerts no significant influence under the tested conditions.

## 4. Discussion

This study further establishes the design characteristics of the modular KWC/GRB vaccine platform (Figure 1) [12]. Using the HIV-1 FP, a conserved target of broadly neutralizing HIV mAbs, we demonstrated how a careful DBTL employing iterative modifications to FP-derived Imms presented on the surface GRB can yield dramatic improvements in the immunogenicity of the candidate vaccines. Although the FP has long been recognized as a target of broadly neutralizing antibodies (bn-mAbs) such as VRC34.01, it remains a challenging epitope for HIV vaccine development [54,55,57,58,59,60,84,85,86,87]. Our approach further demonstrates how, using the KWC/GRB platform, a large number of candidate vaccines can be initially designed and then subsequently down-selected for testing in vivo, identifying critical features of Imm design required for maximal immunogenicity.

Our approach extends the prior work by Maeda et al. on our KWC/GRB vaccine platform, which described a simple monomeric PEDV FP vaccine that protected pigs against PEDV challenge but failed to elicit detectable antigen-specific antibodies [12]. In contrast, the present study demonstrated that, when properly engineered for epitope exposure and immune stimulation, the KWC/GRB platform can reproducibly induce strong FP-specific humoral responses. The tight correlation between pre-immunization VRC34.01 binding and post-immunization antibody magnitude (Figure 7 and Figure 8) demonstrates that in vitro antibody binding predicts in vivo immunogenicity, providing a quantitative design-gate within the DBTL workflow [88,89,90,91,92]. This predictive relationship became evident early in the immunization course and strengthened at later time points, indicating that surface accessibility is a major determinant of the evolving humoral response (Section 3.8, Figure 7). In parallel, cross-assay concordance between ELISA and flow–cytometry readouts also emerged as significant as immunization progressed, suggesting that both assays increasingly capture the same underlying biological signal linking antigen display to antibody output (Section 3.9, Figure 8). Together, these findings validate the use of flow–cytometric mAb binding as a quantitative decision-making tool for vaccine triage prior to animal testing—one of the central advantages of a DBTL pipeline.

Several mechanistic insights emerged. Multimeric presentation of FP antigen, from one to five repeats, improved both surface binding and antibody induction (Figure 4), whereas additional FP repeats (8mer or 10mer) produced no additional gain, indicating an Imm repeat limit for optimal antigen density and B-cell engagement [93,94,95,96,97]. Likewise, extending the inter-repeat linker from the short acidic L1 (GDGDG) to the longer α-helical L2 (AQQASSS × 3) significantly enhanced both VRC34.01 binding and antibody induction magnitude (Mann–Whitney U, *p* = 0.0122) (Figure 5g and Figure 9a), experimentally validating the predicted role of semi-rigid linkers in promoting epitope projection and B-cell accessibility on the bacterial surface [97,98,99,100].

Among the immunomodulatory elements, only PADRE demonstrated a statistically significant effect, and exclusively within homomeric vaccines incorporating the L2 linker (Mann–Whitney U, *p* = 0.0159) (Figure 9b). FLIC did not produce measurable differences in antibody magnitude under the conditions tested. Although exploratory analyses initially suggested higher titers in some formulations containing multiple immunomodulatory sequences, these patterns did not reach statistical significance and were not evaluated using factorial models with interaction terms. Accordingly, our interpretation is restricted to context-dependent effects of individual components rather than additive or synergistic contributions. These findings underscore that PADRE can enhance antibody magnitude only in specific structural backgrounds, whereas other immunomodulators did not yield significant effects.

Heteromeric vaccines incorporating distinct FP variants (FP1–FP4) produced the strongest overall combination of surface binding and antibody responses (Figure 6f,g), consistent with broader B-cell recognition [55,101]. Although heteromeric designs exhibited a mild trend toward higher antibody titers relative to homomeric counterparts (*p* = 0.0947), this difference did not reach statistical significance, suggesting that sequence heterogeneity may modestly influence B-cell engagement but remains underpowered for confirmation.

Collectively, these findings illustrate how discrete structural and immunomodulatory variables—particularly linker architecture and PADRE inclusion—quantitatively shape antigen exposure and antibody magnitude. The coherence between ELISA and flow-based endpoints, together with the integrated PCA analyses, supports a model in which Imm accessibility serves as the primary determinant of humoral immune response. These observations underscore the strength of the DBTL cycle in revealing actionable design parameters for rational optimization of bacterial vaccines.

Compared with alternative vaccine platforms—virus-like particles, outer-membrane vesicles, or synthetic nanoparticles—the KWC/GRB system provides an intrinsically adjuvanted, biosafe, and scalable bacterial scaffold [15,16,21,92]. Its advantages include low production cost, ability to use existing bacterin manufacturing infrastructure, and thermostability suitable for global distribution. By presenting Imms in a membrane context, the platform also mimics the native lipid environment required by certain bn-mAbs such as 2F5 targeting the MPER [102,103]. The β-barrel domain of the AT likely facilitates correct folding of displayed Imms, while the bacterial chassis contributes PAMPs that engage innate receptors [12,42,51]. The strong correlation between mAb binding and antibody response (Figure 7b and Figure 8) confirms that antigen accessibility is indispensable for effective humoral induction, reinforcing the use of the genome-reduced bacteria.

In this study, we found that alternative ways of expressing the FP immunogen, as multimers and together with different immunomodulators, yielded vaccines with dramatically different abilities to elicit immune responses. While this work was mainly focused on developing approaches to make better vaccines, it also therefore became clear that the KWC/GRB vaccine technology, with its low cost and rapid production, could also be used to investigate how different protein immunogen features can contribute to immunogenicity. A better understanding of how to increase (or decrease) the immunogenicity of a protein could lead to better fundamental immunological knowledge, as well as enable the development of better vaccines or inform strategies to decrease the potential immunogenicity of protein therapeutics.

Despite inducing high anti-FP antibody titers, no neutralizing activity against HIV-1 pseudoviruses was detected. To contextualize these findings, we integrated AlphaFold pLDDT predictions with antigen-exposure and antibody-response metrics for all constructs (Supplementary File S2—Table S4). The FP region consistently showed low predicted structural confidence (<70, frequently < 50), indicating substantial conformational flexibility in this display context. Although we did not perform structural characterization of the FP displayed on KWC/GRB, prior Env studies indicate that the isolated FP adopts highly flexible, non-native conformations [60,86], providing a mechanistic explanation for the absence of neutralization despite high antibody titers. This outcome reflects a limitation of the antigen itself, not of the platform: because the HIV-1 FP does not preserve the structural constraints of the trimeric Env [104,105], it is not well suited for eliciting neutralizing antibodies and therefore should not be interpreted as a test of protective efficacy [84]. In this study, the HIV-1 FP served intentionally as a technically demanding model to identify design principles within a DBTL framework, rather than as a realistic candidate for neutralizing vaccine development. This also underscores that the FP epitope likely requires sequential or combinatorial immunization strategies to drive affinity maturation and orientation-specific antibody responses capable of engaging the native trimeric Env [57,86]. In this regard, combining scaffold-based FP vaccines with trimeric or conformationally stabilized Env immunogens represents a promising path to achieve functional neutralization, as suggested by prior work [57,59,60,87]. The 13 vaccine candidates analyzed here provided adequate power for correlation analyses but were underpowered for multifactor comparisons (Table 5); expanding vaccine diversity and sample size in future DBTL iterations will be important for resolving these trends.

Beyond these antigen-specific limitations, a key strength of this work lies in the KWC/GRB DBTL pipeline itself, which enabled rapid construction, screening, and quantitative evaluation of diverse antigen designs. By integrating structural prediction, in vitro binding, and in vivo immunogenicity, the platform revealed actionable relationships between antigen accessibility and antibody magnitude—highlighting its general utility for rational vaccine optimization.

In summary, this study provides a proof of concept that rational vaccine design—guided by structural prediction, in vitro binding data, and iterative feedback—can predict humoral immunogenicity in our KWC/GRB vaccine platform, which will greatly enhance the design of future Imms to be used in the platform (Figure 2, Figure 7 and Figure 8). Beyond HIV FP, this approach can be generalized to other target Imms from other viral or bacterial pathogens, accelerating translation from design to new safe and effective vaccines.

## 5. Conclusions

This study shows how the KWC/GRB vaccine platform can be systematically improved through a structured Design–Build–Test–Learn (DBTL) process. Using the HIV-1 fusion peptide as a technically challenging model, we identified design features—such as multimeric formats, the extended α-helical linker, and context-dependent inclusion of the PADRE T-cell epitope that can enhance antigen display or antibody responses under specific conditions. We also demonstrated that in vitro VRC34.01 binding predicts the strength of the antibody response in vivo, providing a practical design-gate for selecting candidates before animal testing.

An important limitation of this work is that the HIV fusion peptide does not maintain a native Env-like structure when displayed in isolation. Therefore, although the optimized designs generated strong binding antibodies, neutralizing activity was not expected and was not observed. This limitation means that the present study primarily demonstrates technical optimization of the platform, rather than functional protection. Nonetheless, the DBTL workflow developed here is broadly applicable and can now be applied to antigens with structures more compatible with neutralization, where improvements in antigen display are more likely to translate into biological efficacy.

Overall, our results highlight that combining KWC/GRB with iterative DBTL cycles enables rapid, low-cost, and modular optimization of vaccine designs. When paired with immunogens that preserve native conformations, this platform has strong potential for producing effective next-generation vaccines.

## Figures and Tables

**Table 1:** Fusion peptide (FP) variants used in vaccine immunogens.

FP Variant	Sequence (AA 512–519)
FP1	AVGIGAVF
FP2	AVTIGAVF
FP3	AVGLGAVF
FP4	AVGIGAMF

**Table 2:** HIV-1 pseudoviruses used in neutralization assays and correspondence to FP 355 immunogen variants.

Virus	Sequence (AA 512–519)	Tier (Clade)	Corresponding FP Immunogen Variant
25710-2.43	AVGIGAVF	2 (C)	FP1
BJOX002000.03.2	AVGIGAVF	2 (B)	FP1
CH119.10	AVGIGAVF	2 (B)	FP1
MN.3	RAAIGALF	1A (B)	None

**Table 3:** Components of the vaccine candidates.

Component	Name	Sequence	Abbreviation	Function	Refs.
Motif	DADADA Box	DADADA	3DA	Increases expression of AT-expressed recombinant passenger proteins	NA
Antigens	Fusion Peptide 1	AVGIGAVF	FP1	HIV target	[54,58]
	Fusion Peptide 2	AVTIGAVF	FP2	HIV target	
	Fusion Peptide 3	AVGLGAVF	FP3	HIV target	
	Fusion Peptide 4	AVGIGAMF	FP4	HIV target	
Immunomodulators	Flagellin from Salmonella enterica	AQVINTNSLSLLTQNNLNKS QSALGTAIERLSSGLRINSAK DDAAGQAIANRFTANIKGL TQASRNANDGISIAQTTEGA LNEINNNLQRVRELAVQ SA	FLIC	TLR5 agonist	[64,65,66,83] 12/17/2025 10:30:00 AM
	Pan DR Epitope non-cognate T-cell antigen	AKYVAAWTLKAAA	PADRE	Enhances the adaptive immune response by providing broad T-helper cell epitope coverage, thereby improving T-cell activation and memory	[11,67,68]
	Grp A Strep Recombinant Surface Immunogenic Protein	ASVASVQAQETDTTWTARTV SEVKADLVKQDNKSSYTVKY GDTLSVISEAMSIDMNVLAKI NNIADINLIYPETTLTVTYDQ KSHTATSMKIETPATNAAGQ TTATVDLKTNQVSVADQKV	rSIP	TLR2 and 4 agonists	[17]
	Mastoparan	INWKGIAAMAKKLL	MASTO	Enhances immune response by activating immune cells, increasing cell permeability, and triggering local inflammation, promotes interactions with M cells	[69,70,71]
Linkers	Short Linker	GDGDG	L1	Separates the fusion peptides repeats, with additional negatively charged hydrophilic amino acids	NA
	Medium Linker	AQQASSSAQQASSSAQQASSS	L2	Long linker designed to assume a rigid alpha helix to separate the FP units at a distance larger than the size of an immunoglobulin molecule, to promote simultaneous binding of more than one FP unit to B-cell precursors to increase avidity of vaccine binding to a B-cell precursor	NA
	Short Linker	GSGS	L3	Shorter linker with non-charged, hydrophilic amino acids	NA

**Table 4:** Vaccines rounds and compositions.

Round	Large Name	Short Name	Composition
1st	HIV FP 1mer	1mer	3DA + FP1
	HIV FP 5mer GDGDG	5mer L1	3DA + (FP1 + L1)5
2nd	HIV FP HM5mer AQQASSS3	5mer L2	3DA + (L3-FP1-L3 + L2)5
	HIV FP 5mer FLIC AQQASSS3	5mer FLIC L2	3DA + FLIC(L3-FP1-L3 + L2)5
	HIV FP 5mer rSIP AQQASSS3	5mer rSIP L2	3DA + rSIP(L3-FP1-L3 + L2)5
	HIV FP 5mer PADRE AQQASSS3	5mer PADRE L2	3DA + PADRE + (L3-FP1-L3 + L2)5 + PADRE
	HIV FP 8mer FLIC GDGDG	8mer FLIC L1	3DA + (FP1 + L1)4 + FLIC + (FP1 + L1)4
	HIV FP 10mer FLIC AQQASSS3 + GDGDG	10mer FLIC L1/L2	3DA + (L3-FP1-L3 + L2)5 + FLIC + (FP1 + L1)5
3rd	HIV FP HT5mer AQQASSS3	5mer HT L2	3DA + (L3-FP1/FP2/FP3/FP4-L3 + L2)5
	HIV FP HT5mer FLIC AQQASSS3	5mer HT FLIC L2	3DA + FLIC + (L3-FP1/FP2/FP3/FP4-L3 + L2)5
	HIV FP HT5mer PADRE AQQASSS3	5mer HT PADRE L2	3DA + PADRE + (L3-FP1/FP2/FP3/FP4-L3 + L2)5
	HIV FP HT5mer FLIC PADRE AQQASSS3	5mer HT FLIC PADRE L2	3DA + FLIC + PADRE + (L3-FP1/FP2/FP3/FP4-L3 + L2)5
	HIV FP HT5mer MASTOPARAN FLIC PADRE AQQASSS3	5mer HT MASTO FLIC PADRE L2	3DA + MASTO + FLIC + PADRE + (L3-FP1/FP2/FP3/FP4-L3 + L2)5

**Table 5:** Curve-derived metrics from ELISA and flow cytometry data for each vaccine.

Vaccine	Composition	Linker	Immuno- Modulator	Conformation	EC_50__Log10_ELISA	Bmax_ELISA	AUC_ELISA	EC_50__Log10_FLOW	Bmax_FLOW	AUC_FLOW
1mer	1mer	None	None	Homo	11.99	25,942	0.38	0.19	68.44	32.23
5mer L1	5mer	L1	None	Homo	14.08	5199	0.30	−0.57	84.94	84.37
5mer L2	5mer	L2	None	Homo	−1.89	2.31	2.25	−1.52	91.37	166.63
5mer FLIC L2	5mer	L2	FLIC	Homo	−1.72	1.55	1.37	−1.02	88.03	118.99
5mer PADRE L2	5mer	L2	PADRE	Homo	−2.07	2.34	2.66	−0.75	20.43	22.73
5mer rSIP L2	5mer	L2	rSIP	Homo	18.03	28,452	0.36	1.12	335.22	20.11
8mer FLIC L1	8mer	L1	FLIC	Homo	−2.04	0.26	0.32	0.06	81.52	29.57
10mer FLIC L1/L2	10mer	L1 + L2	FLIC	Homo	−2.19	0.49	0.65	0.05	77.49	32.93
5mer HT L2	5mer	L2	None	Hetero	−2.25	2.32	2.99	−1.92	93.40	209.57
5mer HT FLIC L2	5mer	L2	FLIC	Hetero	0.33	9.61	2.40	−2.11	99.33	181.45
5mer HT PADRE L2	5mer	L2	PADRE	Hetero	0.26	9.65	3.20	−2.19	93.84	229.65
5mer HT FLIC PADRE L2	5mer	L2	FLIC + PADRE	Hetero	−1.41	2.89	2.00	−1.78	81.17	161.91
5mer HT MASTO FLIC PADRE L2	5mer	L2	MASTO + FLIC + PADRE	Hetero	−1.18	3.46	1.90	−0.97	85.24	117.53

Note: EC_50_ values are log_10_-transformed. For flow data, exposure_score = −log_10_(EC_50_) (higher = better binding).

## Data Availability

The data of this study are available upon reasonable request from the authors.

## Supplementary Materials

The following supporting information can be downloaded at: https://www.mdpi.com/article/10.3390/vaccines14010014/s1, Supplementary File S1: Plasmid DNA sequences (.gb). Supplementary File S2: Raw experimental data (Excel workbook; .xlsx) containing: Table S1: Raw flow cytometry data (MFI vs. dilution curves for all constructs). Table S2: Raw ELISA data (optical density vs. serum dilution for all animals and timepoints). Table S3: Raw neutralization data (RLU values, percent neutralization, and ID50/ID80 determinations). Table S4: AlphaFold pLDDT values for the FP region, antigen-exposure metrics (AUC_FLOW), humoral-response magnitude (AUC_ELISA), and neutralization outcomes (ID50) for all FP-based immunogens. Supplementary File S3: R scripts for data processing, curve fitting, and statistical testing (.R).

## Abbreviations

The following abbreviations are used in this manuscript:

3DA	DADADA motif
4PL	Four-parameter logistic
AF488	Alexa Fluor 488
AIDA-I	Adhesin Involved in Diffuse Adherence I
ARRIVE	Animal Research: Reporting of In Vivo Experiments
AT/ATs	Autotransporter(s)
AUC_ELISA	Area under the ELISA curve
AUC_FLOW	Area under the flow–cytometry curve
Bmax	Maximum binding (ELISA)
bn-mAbs	Broadly neutralizing monoclonal antibody
BSA	Bovine Serum Albumin
BSL-2	Biosafety Level 2
CDC	Centers for Disease Control and Prevention
CFU	Colony-Forming Units
CI/CIs	Confidence interval(s)
DBTL	Design–Build–Test–Learn
DTP	Diphtheria–Tetanus–Pertussis
EC_50_	Half-maximal effective concentration
ELISA	Enzyme-Linked Immunosorbent Assay
Env	Envelope glycoprotein (HIV)
FBS	Fetal Bovine Serum
FLIC	Flagellin fragment (TLR5 agonist)
FLOW	Flow–cytometry readout (context of manuscript)
FP	Fusion Peptide
FP1–FP4	Fusion Peptide variants 1–4
GDGDG	Acidic linker sequence
GGG	Glycine-repeat linker sequence
GRB	Genome-Reduced Bacteria
HBSS	Hank’s Balanced Salt Solution
HET3	Outbred HET3 mouse stock
HIV/HIV-1	Human Immunodeficiency Virus/type 1
HM	Homomeric (in vaccine names)
HRP	Horseradish Peroxidase
HT	Heteromeric (in vaccine names)
IBC	Institutional Biosafety Committee
ID50/ID80	50%/80% inhibitory dilution
IgG	Immunoglobulin G
IM	Intramuscular
Imm/Imms	Immunogen/Immunogens
L/L1/L2	Linkers (L1 = GDGDG; L2 = AQQASSS × 3)
LB	Lysogeny Broth (Luria–Bertani)
LMIC(s)	Low- and Middle-Income Countries
LPS	Lipopolysaccharide
mAb	Monoclonal antibody
mRNA	Messenger RNA
MPER	Membrane-Proximal External Region
NIAID	National Institute of Allergy and Infectious Diseases
OD450/OD600	Optical density at 450/600 nm
PCA	Principal Component Analysis
PC1	Principal Component 1
PBS	Phosphate-Buffered Saline
PEDV	Porcine Epidemic Diarrhea Virus
pLDDT	Predicted Local Distance Difference Test (score)
pTM	Predicted TM-score
RAU	Relative Antibody Units
RLU	Relative Light Units
rSIP	(Group B streptococcal) surface immunogenic protein
L3	Short flexible linker
SOC	Super Optimal broth with Catabolite repression
SOP(s)	Standard Operating Procedure(s)
TAE	Tris–Acetate–EDTA
TBST	Tris-Buffered Saline with Tween-20
Tfh	T follicular helper (cells)
TMB	3,3′,5,5′-Tetramethylbenzidine
TLR	Toll-Like Receptor
TZM-bl	Reporter cell line for HIV neutralization
UVA	University of Virginia
UV	Ultraviolet
UniProt	Universal Protein Resource (database)
KWC/GRB	Killed Whole-Cell genome-reduced Bacteria
KWC	Killed Whole-Cell
*E. coli*	*Escherichia coli*
