# Supplementary material for: Engineering Enhanced Immunogenicity of Surface-Displayed Immunogens in a Killed Whole-Cell Genome-Reduced Bacterial Vaccine Platform Using Class I Viral Fusion Peptides
Source: Vaccines (Basel). 2025 Dec 22;14(1):14. doi: 10.3390/vaccines14010014 (PMC12846404; doi:10.3390/vaccines14010014)
Supplement: Supplementary file 1 [file vaccines-14-00014-s001.zip › Supplementary_File_S2.pdf]

Table S1. Raw flow cytometry data

| Dilution | Binding | Vaccine |
|---|---|---|
| 0 | 0 | 1mer |
| 0.0017 | 0 | 1mer |
| 0.0035 | 0 | 1mer |
| 0.007 | 0 | 1mer |
| 0.015 | 0 | 1mer |
| 0.03 | 2 | 1mer |
| 0.07 | 5 | 1mer |
| 0.15 | 10 | 1mer |
| 0.3 | 15 | 1mer |
| 0.6 | 22 | 1mer |
| 1.25 | 30 | 1mer |
| 2.5 | 40 | 1mer |
| 0 | 0 | 5mer L1 |
| 0.0017 | 0 | 5mer L1 |
| 0.0035 | 0 | 5mer L1 |
| 0.007 | 0 | 5mer L1 |
| 0.015 | 0 | 5mer L1 |
| 0.03 | 3 | 5mer L1 |
| 0.07 | 7 | 5mer L1 |
| 0.15 | 25 | 5mer L1 |
| 0.3 | 42 | 5mer L1 |
| 0.6 | 75 | 5mer L1 |
| 1.25 | 80 | 5mer L1 |
| 2.5 | 82 | 5mer L1 |
| 0 | 0 | 5mer L2 |
| 0.0017 | 0 | 5mer L2 |
| 0.0035 | 0 | 5mer L2 |
| 0.007 | 5 | 5mer L2 |
| 0.015 | 20 | 5mer L2 |
| 0.03 | 50 | 5mer L2 |
| 0.07 | 62 | 5mer L2 |
| 0.15 | 81 | 5mer L2 |
| 0.3 | 85 | 5mer L2 |
| 0.6 | 87 | 5mer L2 |
| 1.25 | 90 | 5mer L2 |
| 2.5 | 95 | 5mer L2 |
| 0 | 0 | 5mer FLIC L2 |
| 0.0017 | 0 | 5mer FLIC L2 |
| 0.0035 | 0 | 5mer FLIC L2 |
| 0.007 | 1 | 5mer FLIC L2 |
| 0.015 | 9 | 5mer FLIC L2 |
| 0.03 | 19 | 5mer FLIC L2 |
| 0.07 | 38 | 5mer FLIC L2 |
| 0.15 | 50 | 5mer FLIC L2 |
| 0.3 | 65 | 5mer FLIC L2 |
| 0.6 | 75 | 5mer FLIC L2 |
| 1.25 | 80 | 5mer FLIC L2 |
| 2.5 | 85 | 5mer FLIC L2 |
| 0 | 0 | 5mer PADRE L2 |
| 0.0017 | 0 | 5mer PADRE L2 |
| 0.0035 | 0 | 5mer PADRE L2 |
| 0.007 | 0 | 5mer PADRE L2 |
| 0.015 | 1 | 5mer PADRE L2 |
| 0.03 | 2 | 5mer PADRE L2 |

| | | |
|---|---|---|
| 0.07 | 5 | 5mer PADRE L2 |
| 0.15 | 10 | 5mer PADRE L2 |
| 0.3 | 13 | 5mer PADRE L2 |
| 0.6 | 15 | 5mer PADRE L2 |
| 1.25 | 17 | 5mer PADRE L2 |
| 2.5 | 20 | 5mer PADRE L2 |
| 0 | 0 | 5mer rSIP L2 |
| 0.0017 | 0 | 5mer rSIP L2 |
| 0.0035 | 0 | 5mer rSIP L2 |
| 0.007 | 0 | 5mer rSIP L2 |
| 0.015 | 0 | 5mer rSIP L2 |
| 0.03 | 0.5 | 5mer rSIP L2 |
| 0.07 | 1 | 5mer rSIP L2 |
| 0.15 | 2 | 5mer rSIP L2 |
| 0.3 | 5 | 5mer rSIP L2 |
| 0.6 | 11 | 5mer rSIP L2 |
| 1.25 | 23 | 5mer rSIP L2 |
| 2.5 | 46 | 5mer rSIP L2 |
| 0 | 0 | 8mer FLIC L1 |
| 0.0017 | 0 | 8mer FLIC L1 |
| 0.0035 | 0 | 8mer FLIC L1 |
| 0.007 | 0 | 8mer FLIC L1 |
| 0.015 | 0 | 8mer FLIC L1 |
| 0.03 | 0 | 8mer FLIC L1 |
| 0.07 | 0.5 | 8mer FLIC L1 |
| 0.15 | 1 | 8mer FLIC L1 |
| 0.3 | 2 | 8mer FLIC L1 |
| 0.6 | 6 | 8mer FLIC L1 |
| 1.25 | 48 | 8mer FLIC L1 |
| 2.5 | 78 | 8mer FLIC L1 |
| 0 | 0 | 10mer FLIC L1/L2 |
| 0.0017 | 0 | 10mer FLIC L1/L2 |
| 0.0035 | 0 | 10mer FLIC L1/L2 |
| 0.007 | 0 | 10mer FLIC L1/L2 |
| 0.015 | 0 | 10mer FLIC L1/L2 |
| 0.03 | 1 | 10mer FLIC L1/L2 |
| 0.07 | 2 | 10mer FLIC L1/L2 |
| 0.15 | 4 | 10mer FLIC L1/L2 |
| 0.3 | 7 | 10mer FLIC L1/L2 |
| 0.6 | 15 | 10mer FLIC L1/L2 |
| 1.25 | 45 | 10mer FLIC L1/L2 |
| 2.5 | 66 | 10mer FLIC L1/L2 |
| 0 | 0 | 5mer HT L2 |
| 0.0017 | 3 | 5mer HT L2 |
| 0.0035 | 11 | 5mer HT L2 |
| 0.007 | 30 | 5mer HT L2 |
| 0.015 | 50 | 5mer HT L2 |
| 0.03 | 70 | 5mer HT L2 |
| 0.07 | 85 | 5mer HT L2 |
| 0.15 | 90 | 5mer HT L2 |
| 0.3 | 91 | 5mer HT L2 |
| 0.6 | 92 | 5mer HT L2 |
| 1.25 | 93 | 5mer HT L2 |
| 2.5 | 94 | 5mer HT L2 |
| 0 | 0 | 5mer HT FLIC L2 |
| 0.0017 | 2 | 5mer HT FLIC L2 |

| | | |
|---|---|---|
| 0.0035 | 7 | 5mer HT FLIC L2 |
| 0.007 | 22 | 5mer HT FLIC L2 |
| 0.015 | 40 | 5mer HT FLIC L2 |
| 0.03 | 60 | 5mer HT FLIC L2 |
| 0.07 | 68 | 5mer HT FLIC L2 |
| 0.15 | 70 | 5mer HT FLIC L2 |
| 0.3 | 75 | 5mer HT FLIC L2 |
| 0.6 | 90 | 5mer HT FLIC L2 |
| 1.25 | 93 | 5mer HT FLIC L2 |
| 2.5 | 94 | 5mer HT FLIC L2 |
| 0 | 0 | 5mer HT PADRE L2 |
| 0.0017 | 8 | 5mer HT PADRE L2 |
| 0.0035 | 20 | 5mer HT PADRE L2 |
| 0.007 | 45 | 5mer HT PADRE L2 |
| 0.015 | 70 | 5mer HT PADRE L2 |
| 0.03 | 80 | 5mer HT PADRE L2 |
| 0.07 | 85 | 5mer HT PADRE L2 |
| 0.15 | 89 | 5mer HT PADRE L2 |
| 0.3 | 94 | 5mer HT PADRE L2 |
| 0.6 | 94 | 5mer HT PADRE L2 |
| 1.25 | 95 | 5mer HT PADRE L2 |
| 2.5 | 96 | 5mer HT PADRE L2 |
| 0 | 0 | 5mer HT FLIC PADRE L2 |
| 0.0017 | 1 | 5mer HT FLIC PADRE L2 |
| 0.0035 | 8 | 5mer HT FLIC PADRE L2 |
| 0.007 | 17 | 5mer HT FLIC PADRE L2 |
| 0.015 | 30 | 5mer HT FLIC PADRE L2 |
| 0.03 | 55 | 5mer HT FLIC PADRE L2 |
| 0.07 | 60 | 5mer HT FLIC PADRE L2 |
| 0.15 | 68 | 5mer HT FLIC PADRE L2 |
| 0.3 | 74 | 5mer HT FLIC PADRE L2 |
| 0.6 | 78 | 5mer HT FLIC PADRE L2 |
| 1.25 | 80 | 5mer HT FLIC PADRE L2 |
| 2.5 | 82 | 5mer HT FLIC PADRE L2 |
| 0 | 0 | 5mer HT MASTO FLIC PADRE L2 |
| 0.0017 | 0 | 5mer HT MASTO FLIC PADRE L2 |
| 0.0035 | 0 | 5mer HT MASTO FLIC PADRE L2 |
| 0.007 | 3 | 5mer HT MASTO FLIC PADRE L2 |
| 0.015 | 8 | 5mer HT MASTO FLIC PADRE L2 |
| 0.03 | 15 | 5mer HT MASTO FLIC PADRE L2 |
| 0.07 | 31 | 5mer HT MASTO FLIC PADRE L2 |
| 0.15 | 50 | 5mer HT MASTO FLIC PADRE L2 |
| 0.3 | 67 | 5mer HT MASTO FLIC PADRE L2 |
| 0.6 | 80 | 5mer HT MASTO FLIC PADRE L2 |
| 1.25 | 81 | 5mer HT MASTO FLIC PADRE L2 |
| 2.5 | 82 | 5mer HT MASTO FLIC PADRE L2 |

Table S2. AUC ELISA Data

| Vaccine | AUC_ELISA Week 3 | AUC_ELISA Week 6 | AUC_ELISA Week 9 |
|---|---|---|---|
| 1mer | 0.2864 | 0.3519 | 0.38 |
| 5mer L1 | 0.2861 | 0.29 | 0.3 |
| 5mer L2 | 0.6882 | 1.5 | 2.25 |
| 5mer FLIC L2 | 1.25 | 1.4 | 1.37 |
| 5mer PADRE L2 | 2.11 | 2.54 | 2.66 |
| 5mer rSIP L2 | 0.3 | 0.4 | 0.36 |
| 8mer FLIC L1 | 0.25 | 0.3 | 0.32 |
| 10mer FLIC L1/L2 | 0.25 | 0.7 | 0.65 |
| 5mer HT L2 | 0.7765 | 2.077 | 2.99 |
| 5mer HT FLIC L2 | 0.61 | 1.79 | 2.4 |
| 5mer HT PADRE L2 | 0.43 | 1.85 | 3.2 |
| 5mer HT FLIC PADRE L2 | 0.57 | 1.64 | 2 |
| 5mer HT MASTO FLIC PADRE L2 | 0.56 | 1.1 | 1.9 |

Table S3. Neutralization titer

| Vaccine | Week | **25710-2.43** | **MN.3** | **BJOX002000.03.2** | **CH119.10** |
|---|---|---|---|---|---|
| 1mer | 0 | 45 | 45 | 45 | 45 |
| 1mer | 9 | 45 | 45 | 45 | 45 |
| 5mer L1 | 0 | 45 | 45 | 45 | 45 |
| 5mer L1 | 9 | 45 | 45 | 45 | 45 |
| 5mer L2 | 0 | 45 | 45 | 45 | 45 |
| 5mer L2 | 9 | 45 | 45 | 45 | 45 |
| 5mer FLIC L2 | 0 | 45 | 45 | 45 | 45 |
| 5mer FLIC L2 | 9 | 45 | 45 | 45 | 45 |
| 5mer PADRE L2 | 0 | 45 | 45 | 45 | 45 |
| 5mer PADRE L2 | 9 | 45 | 45 | 45 | 45 |
| 5mer rSIP L2 | 0 | 45 | 45 | 45 | 45 |
| 5mer rSIP L2 | 9 | 45 | 45 | 45 | 45 |
| 8mer FLIC L1 | 0 | 45 | 45 | 45 | 45 |
| 8mer FLIC L1 | 9 | 45 | 45 | 45 | 45 |
| 10mer FLIC L1/L2 | 0 | 45 | 45 | 45 | 45 |
| 10mer FLIC L1/L2 | 9 | 45 | 45 | 45 | 45 |
| 5mer HT L2 | 0 | 45 | 45 | 45 | 45 |
| 5mer HT L2 | 9 | 45 | 45 | 45 | 45 |
| 5mer HT FLIC L2 | 0 | 45 | 45 | 45 | 45 |
| 5mer HT FLIC L2 | 9 | 45 | 45 | 45 | 45 |
| 5mer HT PADRE L2 | 0 | 45 | 45 | 45 | 45 |
| 5mer HT PADRE L2 | 9 | 45 | 45 | 45 | 45 |
| 5mer HT FLIC PADRE L2 | 0 | 45 | 45 | 45 | 45 |
| 5mer HT FLIC PADRE L2 | 9 | 45 | 45 | 45 | 45 |
| 5mer HT MASTO FLIC PADRE L2 | 0 | 45 | 45 | 45 | 45 |
| 5mer HT MASTO FLIC PADRE L2 | 9 | 45 | 45 | 45 | 45 |

Table S4. pDDLT value 3D predictions

| Vaccine | AlphaFold pLDDT (FP region) |
|---|---|
| 1mer | < 50 |
| 5mer L1 | < 70 |
| 5mer L2 | < 50 |
| 5mer FLIC L2 | < 50 |
| 5mer PADRE L2 | < 50 |
| 5mer rSIP L2 | < 50 |
| 8mer FLIC L1 | < 70 |
| 10mer FLIC L1/L2 | < 60 |
| 5mer HT L2 | < 50 |
| 5mer HT FLIC L2 | < 50 |
| 5mer HT PADRE L2 | < 50 |
| 5mer HT FLIC PADRE L2 | < 50 |
| 5mer HT MASTO FLIC PADRE L2 | < 50 |
